# Basal and cold-induced fatty acid uptake of human brown adipose tissue is impaired in obesity

**DOI:** 10.1038/s41598-020-71197-2

**Published:** 2020-09-01

**Authors:** T. J. Saari, J. Raiko, M. U-Din, T. Niemi, M. Taittonen, J. Laine, N. Savisto, M. Haaparanta-Solin, P. Nuutila, K. A. Virtanen

**Affiliations:** 1 Turku PET Centre, Turku University Hospital, Kiinamyllynkatu 4-8, 20520 Turku, Finland; 2 Turku PET Centre, University of Turku, Kiinamyllynkatu 4-8, 20520 Turku, Finland; 3 Department of Surgery, Turku University Hospital, Kiinamyllynkatu 4-8, 20520 Turku, Finland; 4 Department of Anesthesiology, Turku University Hospital, Kiinamyllynkatu 4-8, 20520 Turku, Finland; 5 Department of Pathology, Turku University Hospital, Kiinamyllynkatu 4-8, 20520 Turku, Finland; 6 MediCity Research Laboratories, University of Turku, Tykistökatu 6A, 20520 Turku, Finland; 7 Institute of Public Health and Clinical Nutrition, University of Eastern Finland, PL 1627, 70211 Kuopio, Finland

**Keywords:** Obesity, Adipocytes, Fat metabolism, Obesity

## Abstract

Fatty acids (FA) are important substrates for brown adipose tissue (BAT) metabolism, however, it remains unclear whether there exists a difference in FA metabolism of BAT between lean and obese healthy humans. In this study we evaluated supraclavicular BAT fatty acid uptake (FAU) along with blood perfusion in lean and obese subjects during cold exposure and at room temperature using positron emission tomography (PET)/computed tomography (CT). Additionally, tissue samples were taken from supraclavicular region (typical BAT region) from a subset of subjects to evaluate histological presence of BAT. Non-shivering cold stress elevated FAU and perfusion of BAT in lean, but not in obese subjects. Lean subjects had greater FAU in BAT compared to obese subjects during cold exposure and interestingly also at room temperature. The higher BAT FAU was related to younger age and several indicators of superior systemic metabolic health. The subjects who manifested BAT histologically had several folds higher BAT FAU compared to subjects with no such histological manifestation. Together, obese subjects have less active tissue in supraclavicular region both in basal and cold-activated state and the FA metabolism of BAT is blunted in obesity.

## Introduction

Brown adipose tissue (BAT) is specialized in producing heat. For thermogenesis it utilizes intracellular stored triglycerides as well as circulating free fatty acids (FFA) and glucose^1^. The cold stress elevates BAT metabolism for UCP-1 mediated thermogenesis effectively: the tissue is activated to maintain body temperature without muscle shivering known as non-shivering thermogenesis (NST)^2^. In human studies where accumulation of radiolabeled glucose analogue ^18^F-2-fluoro-2-deoxy-d-glucose (^18^F-FDG) has been used as a marker of thermogenesis, it was shown that BAT glucose uptake increases in cold conditions^3–5^. In addition to higher glucose uptake, the blood perfusion of BAT has found to be increased in response to cold exposure^3,6^. BAT is also considered to have role in the energy balance of adult humans; it has been shown that cold-induced blood flow and glucose uptake are remarkably lower in obese subjects than in lean subjects^7,8^.

While glucose metabolism of BAT has been more widely studied, the role of fatty acids in human BAT metabolism is not yet clear. In rodents BAT relies on fatty acids up to 90% over glucose to generate heat^1^. The primary source of fatty acids for oxidation in BAT is thought to be the intracellular lipid pool^1,9,10^. If the fatty acids derived from intracellular stores are not available, circulatory glucose and free fatty acids derived from white adipose tissue lipolysis become important sources of energy for BAT^11,12^. It has been shown that the thermogenic capacity of human BAT is reduced when intracellular triglyceride lipolysis is suppressed, supporting the role intracellular lipids have in BAT function^9^. Circulatory fatty acid uptake (FAU) of BAT has been studied with positron emission tomography (PET) imaging using 14(R,S)-^18^F-fluoro-6-thia-heptadecanoic acid (^18^F-FTHA) as radiotracer^13,14^. ^18^F-FTHA is a palmitate analogue with high organ to plasma input radioactivity ratio, which allows for reliable PET quantification of tissues specific uptake^15^. The method of measuring fatty acid uptake in BAT using ^18^F-FTHA may represent the uptake of circulatory free fatty acid (FFA) as well as the lipoprotein lipase (LPL) mediated release of fatty acid from the hydrolysis of triglycerides. Once ^18^F-FTHA has been injected, roughly half of it undergoes glycerol ester formation, as it has been noted that post ^18^F-FTHA injection the levels of ^18^F-FTHA labelled plasma triglycerides increases^15^. Previously, cold stimulated uptake of ^18^F-FTHA in BAT has been studied in a small population, but no comparison to basal room temperature measurements have been made^16^. Additionally, the comparison of FAU in cold and in ambient room temperature has not been done, limiting our present understanding of fatty acid metabolism of BAT in cold-stimulated and basal state.

It has been argued that there may be a connection between inactive BAT and obesity and related metabolic disorders^7^. The cause of reduced BAT activity in obese subjects is not yet clear, however it has been shown that weight loss after bariatric surgery increases BAT FAU^13^.

**Figure 1:**
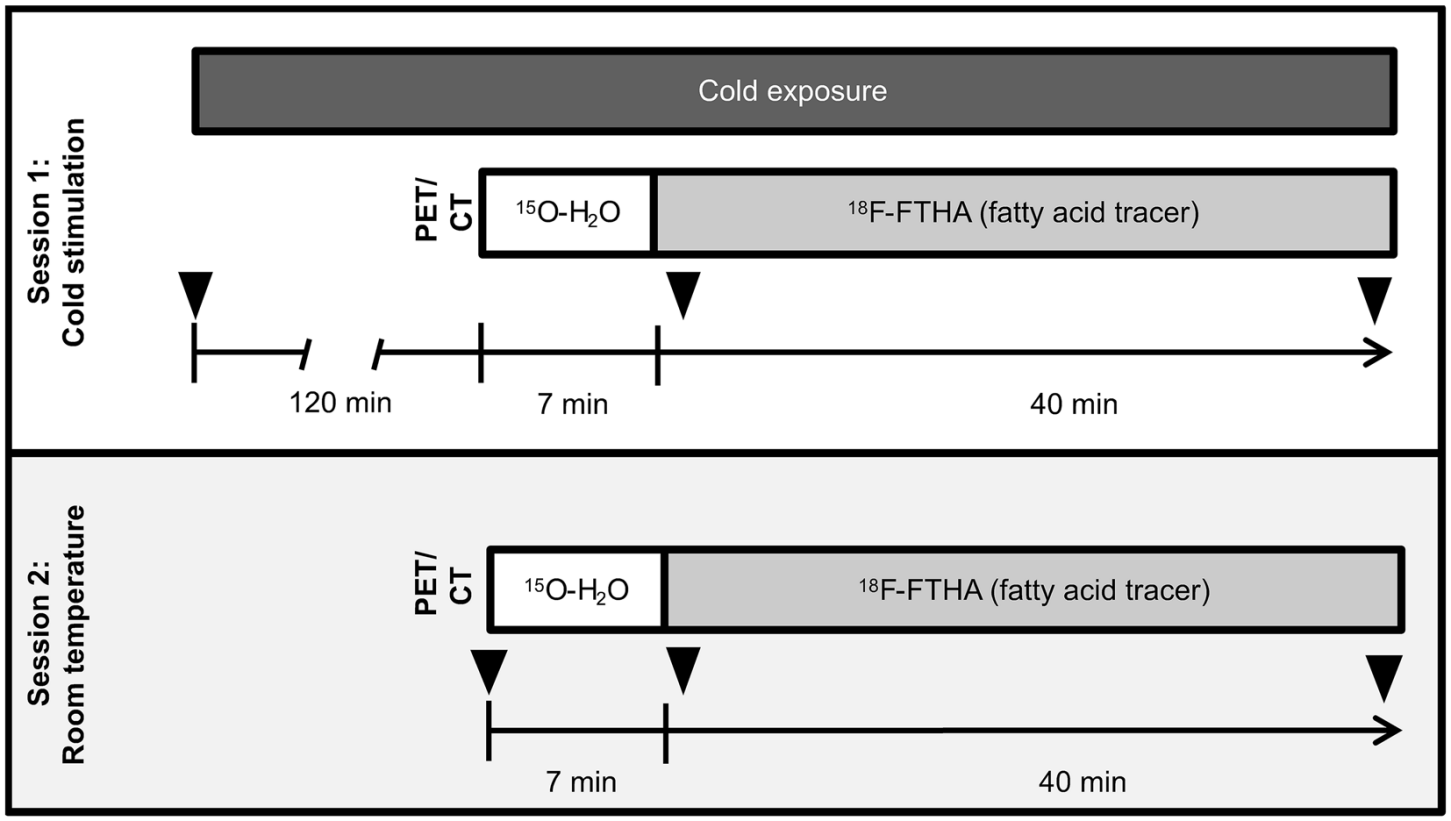
PET/CT Study design represented as a timeline. All subjects were scanned after an overnight fast. Subjects underwent two-hours of cold exposure before PET session (Session 1) and cold stimulation was continued throughout scanning (n = 39). A subset of the study participants also underwent another PET/CT scan without cold exposure in room temperature conditions (Session 2), on a separate study visit. Both scanning protocols were similar, with the exception of cold exposure; subjects were scanned with CT and afterwards with PET using ^15^O-H_2_O and ^18^F-FTHA. Triangles indicate times when blood samples were drawn during the scans.

**Figure 2:**
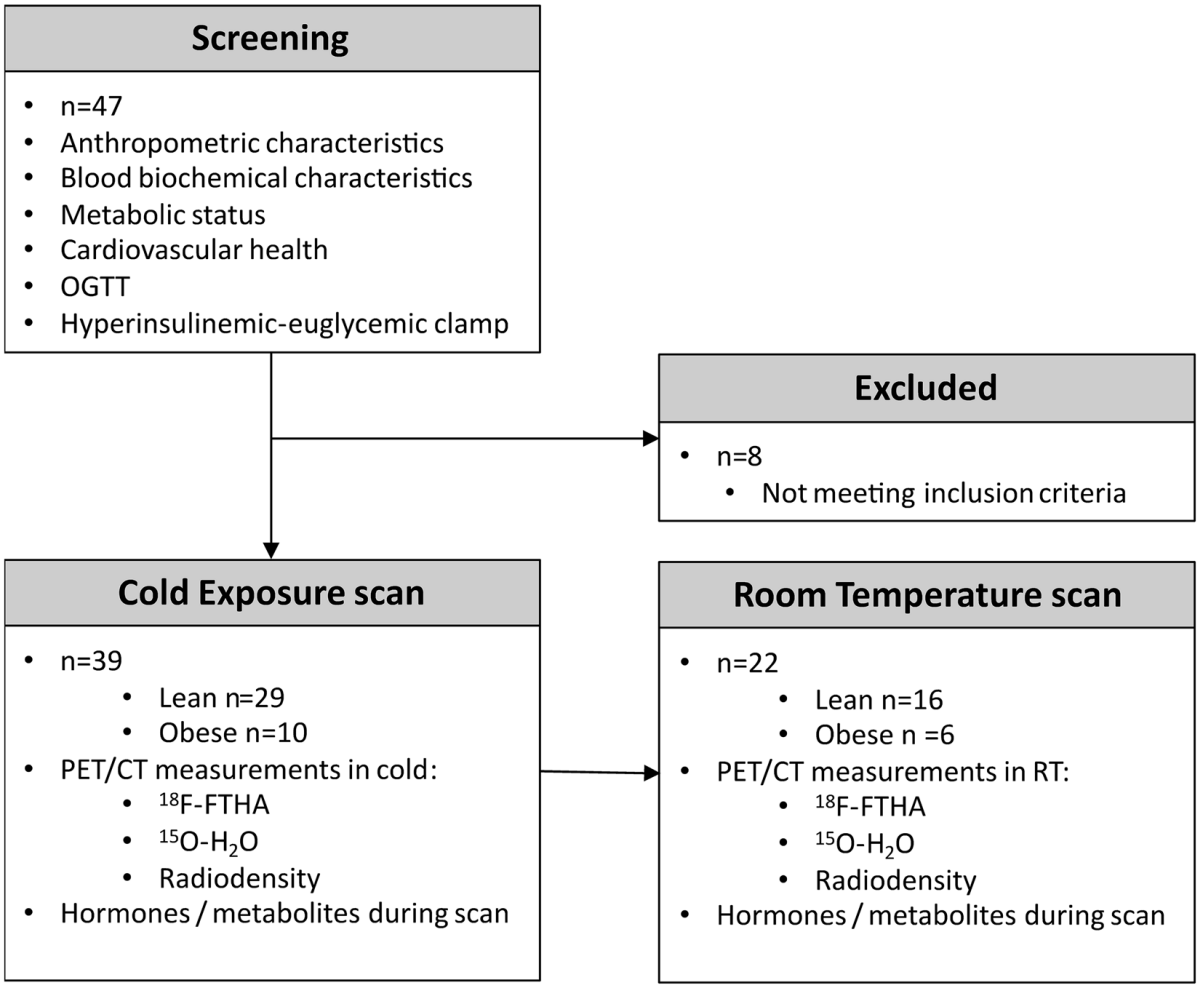
Outline of study subjects and measurements taken during screening and PET/CT scans. Metabolic and cardiovascular health was assessed before enrollment into the study. In addition to cold exposure scans, a subset of subjects were imaged at room temperature. More detailed group allocation and exclusions from analysis can be found in Supplementary Figure 1.

This study was designed to improve our current understanding of fatty acid metabolism of BAT in adult humans and the possible effects obesity may have on BAT metabolism. Our hypothesis was that cold exposure increases BAT FAU in lean subjects, but not in obese subjects. We quantified FAU of BAT with PET imaging using a palmitate analogue ^18^F-FTHA and tissue perfusion using ^15^O-H_2_O. Additionally, computed tomography (CT) was used, in conjunction with PET imaging, as an anatomical reference imaging and as a method to give insight to intracellular lipid metabolism of BAT^17^. BAT metabolism was studied during acute non-shivering cold stress and at room temperature, in order to compare BAT metabolism during cold stimulatory and at basal state (Fig. 1). Both lean and obese subjects (Fig. 2) were included to determine whether obesity has an influence on fatty acid metabolism of BAT.

## Results

Baseline measurements and anthropometric data is available in Table 1. We measured FAU in supraclavicular BAT, subcutaneous white adipose tissue (WAT) and deltoid muscle (Table 2). The tissues measured beside BAT were to estimate whether they contribute significantly to an increased whole-body fatty acid utilization during cold exposure^18^. Exposure to cold caused an increase of FAU only in the BAT region (p = 0.018), while no differences were observed in WAT (p = 0.66), or in deltoid muscle (p = 0.213).

**Table 1:** General characteristics and comparison of lean and obese subjects and measurements taken at baseline, and during cold exposure or room temperature scans.

	Lean subjects n = 29	Obese subjects n = 10	p-value
Age (years)	36.60 ± 10.50	42.40 ± 8.68	0.10
Gender (M/F)	18F/11M	6F/4M	
BMI (kg/m^2^)	25.09 ± 2.47	33.56 ± 3.36	0.0001
Waist circumference (cm)	88.04 ± 13.10	106.45 ± 16.65	0.007
M-value (mg/kg/min)	8.38 ± 2.85	5.64 ± 3.87	0.03
Matsuda index	6.05 ± 2.85	7.34 ± 2.51	0.22
HOMA-IR	2.01 ± 0.89	1.54 ± 0.77	0.16
Insulinogenic index	0.88 ± 0.48	0.85 ± 0.43	0.84
Fat percentage of weight (%)	30.08 ± 8.18	38.61 ± 7.72	0.009
HDL cholesterol (mmol/L)	1.64 ± 0.39	1.43 ± 0.32	0.15
LDL cholesterol (mmol/L)	2.76 ± 0.79	2.84 ± 0.73	0.80
Total cholesterol (mmol/L)	4.62 ± 0.91	4.93 ± 0.97	0.45
Triglycerides (mmol/L)	0.79 ± 0.29	1.41 ± 0.97	0.11
HbA_1c_ (mmol/mol)	35.01 ± 4.43	31.91 ± 5.63	0.97
Alanine transaminase (U/L)	27.89 ± 21.46	24.88 ± 20.34	0.72
Gamma-glutamyltransferase (U/L)	24.83 ± 22.14	26.75 ± 25.43	0.85
Alkaline phosphatase (U/L)	62.48 ± 19.66	53.13 ± 13.04	0.13
**Cold exposure**			
BAT radiodensity (HU)	− 82 ± 6	− 92 ± 8	0.0001
WAT radiodensity (HU)	− 83.03 ± 6.32	− 83.86 ± 5.21	0.69
Deltoid radiodensity (HU)	58.76 ± 19.36	13.61 ± 29.67	0.001
**Room tempeature**			
BAT radiodensity (HU)	− 84 ± 5	− 94 ± 8	0.0003
WAT radiodensity (HU)	− 84.11 ± 4.76	− 86.14 ± 3.61	0.30
Deltoid radiodensity (HU)	53.19 ± 19.78	22.94 ± 28.47	0.009

Values are presented as mean ± SD.

Unpaired student t-test has been used to compare data between lean and obese study subjects.

**Table 2:** Pairewise comparison of measurements taken during cold exposure or room temperature scans of lean and obese subjects.

	Cold exposure	Room temperature	p-value
**Lean subjects** **n = 16**			
BAT fatty acid uptake (µmol/100 g/min)	1.15 ± 1.05	0.64 ± 0.45	0.025
BAT blood flow (mL/100 g/min)	12.50 ± 5.00	7.52 ± 4.64	0.039
BAT radiodensity (HU)	− 83.16 ± 6.30	− 85.27 ± 5.54	0.001
WAT fatty acid uptake (µmol/100 g/min)	0.28 ± 0.11	0.25 ± 0.09	0.32
WAT blood flow (mL/100 g/min)	2.93 ± 1.68	2.50 ± 1.19	0.423
Deltoid fatty acid uptake (µmol/100 g/min)	0.52 ± 0.17	0.45 ± 0.12	0.134
Deltoid blood flow (mL/100 g/min)	2.75 ± 2.30	2.11 ± 1.22	0.371
**Obese subjects** **n = 6**			
BAT fatty acid uptake (µmol/100 g/min)	0.35 ± 0.10	0.25 ± 0.13	0.36
BAT blood flow (mL/100 g/min)	9.53 ± 4.64	6.14 ± 5.10	0.308
BAT radiodensity (HU)	− 94.22 ± 9.04	− 95.92 ± 9.10	0.073
WAT fatty acid uptake (µmol/100 g/min)	0.19 ± 0.05	0.24 ± 0.16	0.408
WAT blood flow (mL/100 g/min)	3.45 ± 1.64	4.87 ± 5.11	0.461
Deltoid fatty acid uptake (µmol/100 g/min)	0.36 ± 0.08	0.37 ± 0.13	0.952
Deltoid blood flow (mL/100 g/min)	2.92 ± 2.62	2.28 ± 1.31	0.451

Values are presented as mean ± SD.

Paired t-test has been used to compare data between lean and obese study subjects.

Additionally, we also measured the differences in tissue specific blood perfusion rates. The cold stress stimulated an increase in tissue perfusion of BAT (p = 0.018), while no differences were seen in tissue perfusion of WAT (p = 0.873) and deltoid muscle (p = 0.243).

We also observed an increase in CT radiodensity, indicated by Hounsfield unit (HU), of BAT region in cold exposure (p < 0.001). This effect was not seen in deltoid muscle (p = 0.52) or WAT (p = 0.68).

**Figure 3:**
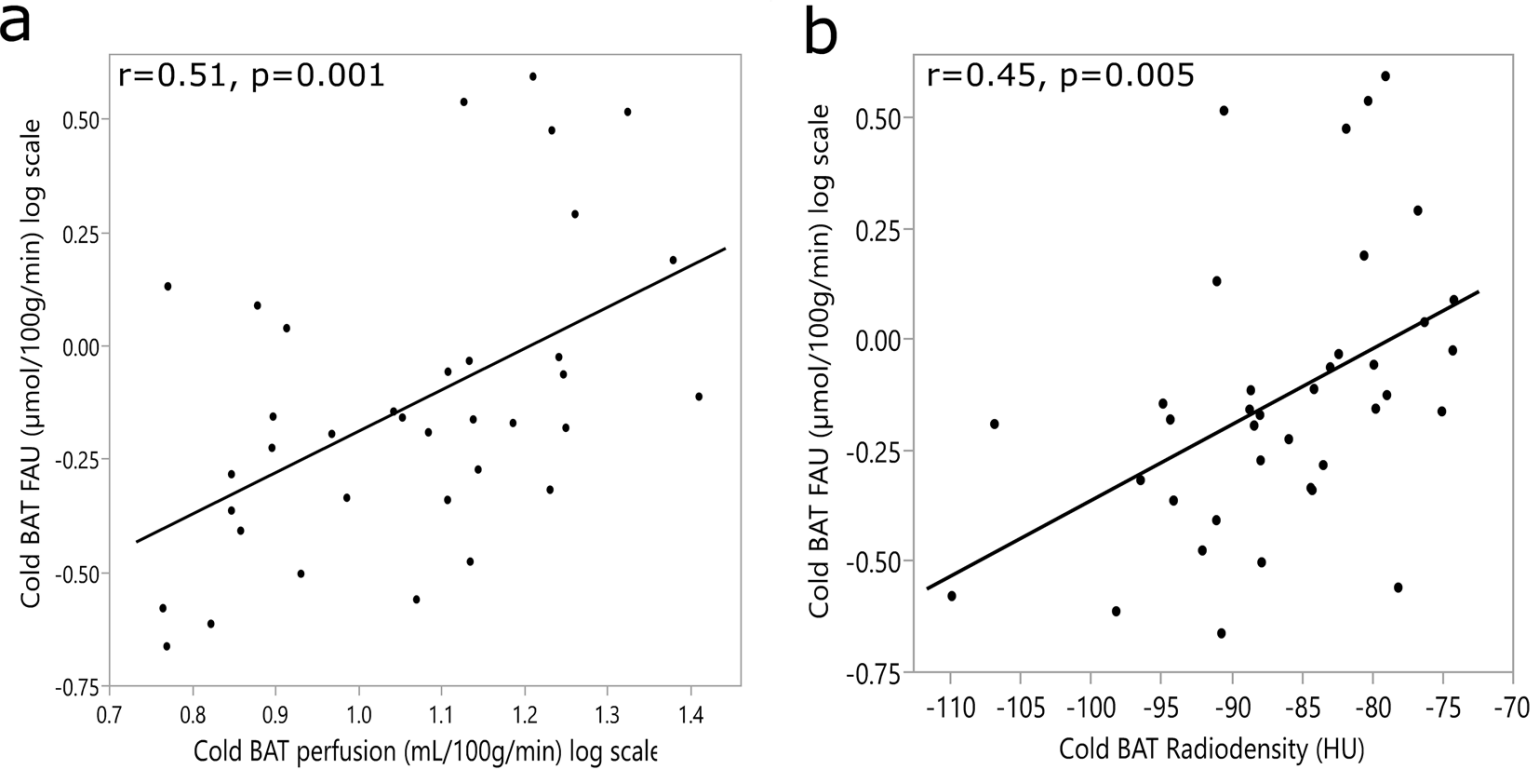
Pearson correlations between measurements taken during cold exposure. (**a**) Subjects with higher BAT FAU had higher BAT perfusion (n = 36). (**b**) Subjects with higher BAT radiodensity, indicating a lower tissue stored triglyceride content, had higher BAT FAU (n = 38).

Higher BAT FAU during cold exposure was associated with increased perfusion (r = 0.51, p = 0.001) and radiodensity (r = 0.45, p = 0.005) during cold exposure (Fig. 3).

Cold stimulated BAT FAU was negatively associated with several measured variables related to obesity; waist circumference (r = − 0.34, p = 0.038), BMI (r = − 0.39, p = 0.015), M-value (r = 0.42, p = 0.01) and age (r = − 0.51, p = 0.001). There was also a positive correlation between BAT FAU and plasma high-density lipoproteins (HDL) values (r = 0.45, p = 0.006).

Using linear regression we found that BAT FAU can be predicted by BAT perfusion (*F*(1, 34) = 11.95, p = 0.001, BAT FAU = − 1.103 + 0.913 × (BAT Perfusion)), and BAT radiodensity (*F*(1, 36) = 9.16, p = 0.005, BAT FAU = 1.344 + 0.017 × (BAT HU)). To assess the relationship of waist circumference, BMI, plasma HDL, age and perfusion to BAT FAU we used multivariate linear regression analysis. This model significantly predicted BAT FAU *F*(5, 27) = 6.60, p < 0.0005, adjusted r^2^ = 0.47. However, only age (p = 0.029) and BAT perfusion (p = 0.043) added statistically to the prediction. Since there was some heterogeneity between our groups, to check for confounding factors, we analyzed the relationships of age, BMI and M-value using multivariate linear regression. Age (p = 0.004) and BMI (p = 0.028) predicted BAT FAU during cold exposure, but M-value was not a significant predictor (p = 0.91) when corrected for age and BMI.

We observed no difference in BAT or WAT FAU or perfusion between male and female subjects in cold or room temperature. Female subjects had higher deltoid muscle FAU (p = 0.01) and perfusion (p = 0.02) compared to male subjects during cold exposure.

### Impaired BAT fatty acid metabolism in obese subjects

**Figure 4:**
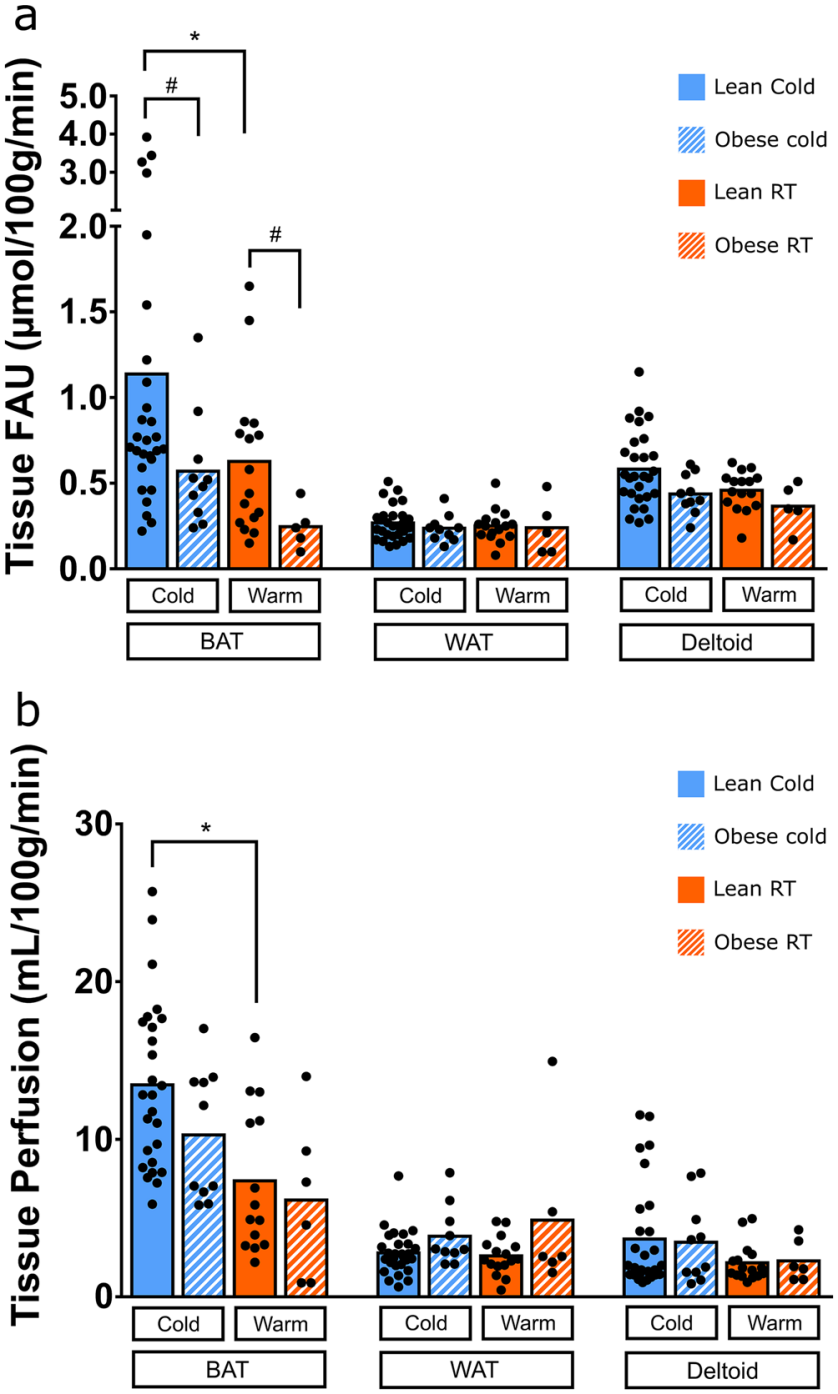
(**a**) Fatty acid uptake rates of BAT, WAT and deltoid muscle measured with ^18^F-FTHA during cold exposure (lean n = 29, obese n = 10) and in room temperature (lean n = 15, obese n = 6). (**b**) Tissue specific perfusion of BAT, WAT, and deltoid muscle measured with ^15^O-H_2_O during cold exposure and room temperature. Results are presented as mean and individual data points. *Indicates p < 0.05 between cold vs. RT in whole group. ^#^Indicates statistical difference of p < 0.05 between lean and obese groups.

When we looked at the effects of obesity on BAT fatty acid metabolism, we observed a blunting effect on FAU in BAT during cold exposure (Fig. 4 and Table 2). BAT FAU in lean subjects was twofold to the obese subjects (p = 0.014). We could see the same effect of obesity to BAT FAU also in the room temperature measurements; the FAU of lean subjects was again twofold to that of the obese subjects (p = 0.006). Deltoid and WAT FAU were similar between lean and obese groups.

In lean subjects, cold stimulation increased BAT FAU (p = 0.025), perfusion (p = 0.039), and CT radiodensity (p = 0.001). While in obese group, we observed that there is no significant response to cold exposure in BAT FAU (p = 0.36), perfusion (p = 0.308) or CT radiodensity (p = 0.073) (Table 2).

### Histologically proven BAT positive subjects

In addition to taking part in PET/CT imaging sessions, a subgroup of study participants (n = 31) volunteered for the biopsy of the supraclavicular fat depot. Morphology of supraclavicular biopsies was studied by a pathologist and subjects with tissue resembling BAT were designated as BAT^(+)^, subjects with no tissue resembling BAT were designated BAT^(−)^. All in all, seven (4F/3 M) subjects had biopsy proven BAT (Supplementary Table 1).

BAT^(+)^ subjects were younger (27.6 ± 7.7 vs. 40.5 ± 10.7 years, p = 0.004) compared to BAT^(−)^ subjects. However, the groups did not have significantly different BMI (24.8 ± 3.0 vs. 28.1 ± 5.1 kg/m^2^, p = 0.051), waist circumference (85.7 ± 10.9 vs. 95.0 ± 18.9 cm, p = 0.13) or M-value (7.4 ± 2.1 vs. 6.3 ± 3.9 mg/kg/min, p = 0.40). After a point-biserial correlation run between biopsy positive (0) or negative (1) result and BAT FAU and perfusion during cold exposure as well as room temperature, we found a strong negative correlation between negative biopsy result and BAT FAU in cold (r_pb_ = − 0.862, p < 0.001) and room temperature (r_pb_ = − 0.775, p < 0.001) as well as moderate correlation with BAT perfusion in cold (r_pb_ = − 0.384, p = 0.021), but no correlation with room temperature BAT perfusion (r_pb_ = − 0.175, p = 0.8).

**Figure 5:**
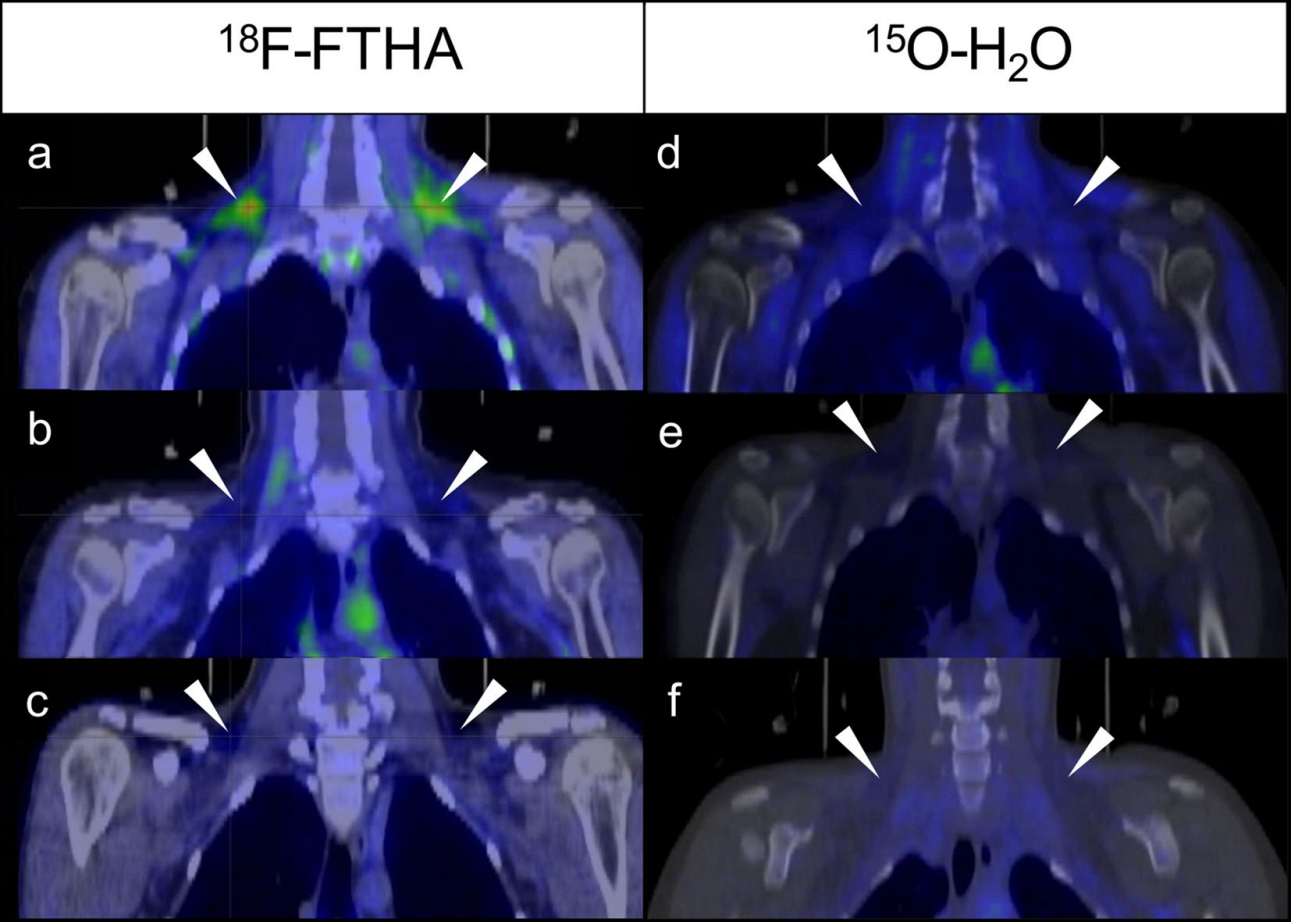
^18^F-FTHA (**a**–**c**) and ^15^O-H_2_O **(d**–**f)** PET/CT fusion images. Triangles indicate location of supraclavicular BAT depots. (**a**) BAT^(+)^ subject in cold exposure. Symmetrical activation of BAT can be seen in the supraclavicular adipose deposits. (**b**) BAT^(+)^ subject in room temperature. (**c**) Lean BAT^(−)^ subject in cold exposure. No activity in the supraclavicular region. (**d**) BAT^(+)^ subject in cold exposure, showing increased perfusion in supraclavicular area. (**e**) Same BAT^(+)^ subject as in image D in room temperature, minor supraclavicular activity. (**f**) BAT^(−)^ subject in cold exposure.

BAT^(+)^ subjects had threefold cold stimulated BAT FAU compared to the lean BAT^(−)^ subjects (2.64 ± 1.01 vs. 0.71 ± 0.24 µmol/100 g/min, p = 0.002) and fivefold compared to the obese BAT^(−)^ subjects (2.64 ± 1.01 vs. 0.50 ± 0.22 µmol/100 g/min, p = 0.001). Deltoid muscle FAU was higher in BAT^(+)^ subjects compared to the lean BAT^(−)^ (0.76 ± 0.22 vs. 0.50 ± 0.18 µmol/100 g/min, p = 0.018) and the obese BAT^(−)^ (0.76 ± 0.22 vs. 0.49 ± 0.17 µmol/100 g/min, p = 0.015) groups. WAT FAU was similar in all three groups (BAT^(+)^: 0.31 ± 0.11, Lean BAT^(−)^: 0.25 ± 0.09, Obese BAT^(−)^: 0.24 ± 0.09 µmol/100 g/min). Representative PET/CT-images of BAT^(+)^ and BAT^(−)^ subjects are shown in Fig. 5.

Cold stimulated BAT perfusion was similar between BAT^(+)^ and the lean BAT^(−)^ subjects (16.56 ± 5.80 vs. 12.40 ± 5.09 mL/100 g/min, p = 0.13). Perfusion in the obese BAT^(−)^ was lower compared to BAT^(+)^ subjects (10.61 ± 3.94 vs. 16.56 ± 5.80 mL/100 g/min, p = 0.038). The perfusion of WAT in BAT^(+)^ and the lean BAT^(−)^ groups was similar (2.44 ± 1.21 vs. 3.9 ± 1.9 mL/100 g/min), however the obese BAT^(−)^ subjects had higher WAT perfusion compared to BAT^(+)^ (2.44 ± 1.21 vs. 4.06 ± 1.95 mL/100 g/min, p = 0.031). Deltoid muscle perfusions did not differ between the groups (BAT^(+)^: 1.9 ± 1.9, Lean BAT^(−)^: 1.9 ± 1.1, Obese BAT^(−)^: 3.2 ± 3.9 mL/100 g/min).

### Circulating hormones and metabolites measured during scanning

We measured several circulating metabolites and hormones during the scanning. Plasma FFA levels increased as a result of cold exposure (p < 0.0001), while they remained unchanged during the room temperature scans. Plasma triglyceride levels increased from baseline during cold exposure (p = 0.01) but remained at baseline levels during RT scans. Area under the curve (AUC) of plasma triglycerides during cold exposure scanning was significantly higher compared to that of during the RT scanning (p = 0.04). Plasma Insulin levels decreased from baseline both during cold exposure (p < 0.0001) and room temperature scans (p < 0.0001). During both cold exposure and room temperature scans we observed a decrease of TSH (cold: p = 0.00024, room temperature: p = 0.00017) and T3 (cold: p = 0.029, room temperature: p < 0.0001) hormones over time. However, we did not see significant differences in AUC values, with the exception of plasma triglycerides (Table 3).

**Table 3:** Changes in hormones and metabolites during scanning.

	Cold AUC	Room temperature AUC	ΔAUC	p-value
Skin temperature (°C)	66.20 ± 2.80	68.90 ± 1.19	− 1.78 ± 2.62	0.08
Plasma TSH (mU/L)	2.89 ± 1.18	2.75 ± 1.31	0.15 ± 0.81	0.44
Plasma T3 (mU/L)	8.78 ± 1.07	8.45 ± 1.06	0.34 ± 0.91	0.11
Plasma T4 (mU/L)	26.53 ± 3.57	26.97 ± 4.27	− 0.42 ± 2.52	0.48
Plasma insulin (mU/L)	12.69 ± 7.38	11.51 ± 6.64	0.88 ± 4.59	0.44
Plasma triglycerides (mmol/L)	2.17 ± 0.87	1.78 ± 0.97	0.39 ± 0.77	0.04
Plasma glucose (mmol/L)	10.54 ± 1.22	10.78 ± 0.66	0.19 ± 0.57	0.61
Plasma FFA (mmol/L)	1.58 ± 0.47	1.48 ± 0.40	0.13 ± 0.33	0.12

Expressed as mean ± SD area under curve, calculated from three timepoints of measurement during scanning and difference of AUC between cold and room temperature. P-value from paired t-test between cold and room temperature scans.

We also observed a relationship between plasma triglyceride levels measured during cold exposure and BAT; plasma triglyceride levels at the end of cold exposure correlated negatively with cold-induced BAT FAU levels (r = − 0.34, p = 0.046). We also found an association between BAT HU and plasma triglyceride levels during cold stimulation (r = − 0.54, p = 0.00039).

## Discussion

The fatty acid metabolism of human BAT has not been extensively studied. Our purpose was to investigate circulatory FAU of BAT in activated cold-stimulated state and at basal (room temperature) level in lean and obese subjects, to further enhance our understanding of BAT fatty acid utilization.

With this study, we show that the FAU of brown adipose tissue increases as a result of exposure to cold in lean subjects but not in obese subjects. Lean subjects have two-fold higher FAU in BAT in cold compared to obese subjects. The FAU in BAT measured by our method depicts the uptake of both LPL-mediated FFA released from triglycerides as well as circulatory FFAs^15^. The blood perfusion to BAT is also increased in response to cold in lean subjects, but not in obese subjects. This indicates that obesity blunts the fatty acid metabolism of supraclavicular BAT in the cold-activated state. One explanation to this blunting phenomenon is that the supraclavicular fat depot of obese subjects likely consists mostly of white adipose tissue, or that obese subjects have a blunted ability to activate brown adipose tissue in response to cold exposure. However, interestingly these differences in BAT FAU between lean and obese could also be seen at the basal state: FAU values of lean subjects at basal state (room temperature) were significantly higher compared to the obese subjects. This shows that the obese do not merely have impaired fatty acid metabolism in BAT after cold activation, but this is also evident at room temperature. This observation supports the hypothesis that the differences in cold stimulated glucose uptake and FAU between lean and obese would not be the result of blunted ability to activate BAT, but because of less metabolically active tissue^7^. This is in line with one of our previous studies, where we have shown using ^18^F-FDG PET imaging that obese subjects have limited cold-induced glucose uptake compared to lean^7^. Subjects with histological evidence of BAT have several folds higher FAU in BAT compared to subjects with no histological evidence of BAT. Obesity has also been linked to changes in white adipose tissue metabolism, such as decreased blood flow and glucose uptake rate^19,20^. All substrate utilization of BAT seems to be blunted in obesity, however no differences between lean and obese subjects BAT glucose uptake at basal level have been reported. As a substrate for basal metabolism, it is possible that glucose does not play a prominent role and it is used as a fast source of energy during acute cold stress. A recent report using microdialysis has shown that BAT glucose uptake is greater compared to WAT during room temperature conditions^21^ suggesting that BAT is metabolically active also at warm conditions. We show similar phenomenon in our study: the FAU at room temperature of the supraclavicular fat region is higher compared to WAT in lean subjects, suggesting a presence of relatively active tissue in the supraclavicular region. Additionally, based on these results we recommend the use of ^18^F-FTHA as a PET imaging tracer for the detection of BAT in non-stimulated state.

The reported effect of cold to FAU of BAT (two-fold increase) would seem to be a lower activation when compared to previously reported glucose uptake increases of over tenfold^3,6^. However, it should be taken into account that palmitate yields around three times more ATP per mole compared to glucose, so the potential energy provided by FFAs, subsequent to complete mitochondrial oxidative reactions, during cold exposure is at least on the same level as with glucose. Using ^18^F-FTHA PET/CT it cannot be differentiated whether the taken up fatty acids by the tissue are intracellularly stored or used as energy, since this method measures the uptake of fatty acids. However, BAT FAU has been found to be linked with cellular respiration in cold and in room temperature^18^. Thus, it is likely that differences in fatty acid uptakes in BAT in lean and obese subjects in our report represent diminished oxidative metabolism capacity of BAT in the obese both at basal level and at activated state. Previously it has been reported that glucose uptake, assuming a BAT mass of 150 g, consumes 23 kcal in a day^8^. This was calculated from extrapolated glucose uptake rates measured during cold exposure. If we calculate fatty acid uptake in absolute terms, with the same assumptions, our results show an average uptake rate of 0.20 ± 0.19 g/day with the energy content of 5.16 ± 4.79 kcal/day. Using microdialysis it has been shown that a large portion of glucose taken up by BAT during cold exposure is released as lactate in healthy subjects, which would indicate that most of the glucose taken up is not utilized^21^. It has also been shown that higher BAT radiodensity, indicating lower intracellular triglyceride content, is associated with higher BAT FAU^17^. In this study we also see similar association between BAT radiodensity and FAU, and, we note that BAT HU was negatively associated with plasma triglyceride levels during cold exposure, showing that it is related to cellular respiration and the use of internal triglyceride stores of BAT, further supporting the role of BAT in plasma triglyceride clearance.

BAT glucose metabolism is known to decrease by advancing age and obesity^7,16,22–26^. In line with this, in our study waist circumference and BMI were found to be negatively associated with BAT FAU. Both BMI and age correlated negatively with FAU in the whole studied population. While both BMI and age seem to be limiting factors in BAT fatty acid metabolism, using multivariate regression analysis, we showed that age was more prominent predictor of BAT FAU than BMI. Further, BAT FAU in cold was directly associated with whole-body insulin sensitivity, indicated by M-value, and HDL-cholesterol levels indicating that subjects with high BAT fatty acid metabolism possess lower risk of the metabolic syndrome. We did not observe any difference in BAT activity in cold between male and female subjects. Similar percentage of males and females were BAT^(+)^ and no difference between the genders was seen in BAT FAU or perfusion during cold exposure or room temperature.

We observed a negative relationship between cold activated BAT FAU and measured plasma triglyceride levels during the scan suggesting that higher BAT metabolism may influence circulatory lipid concentrations. This is in line with a study indicating that BAT metabolism is related to plasma triglyceride clearance in rodents^27,28^. We also observed an increase in plasma triglyceride AUC in cold compared to room temperature. It has been shown that after a longer cold exposure, BAT activation was related to decrease in fasting plasma triglycerides and LPL the day after cold exposure^29^. It is possible that there are delayed responses to lipid clearance caused by BAT activation.

A limiting factor in this study was the low number of subjects who had histological evidence of BAT (7 out of 31 that volunteered for biopsy), making statistical comparison difficult. Merely the histology of the tissue samples has been studied, and differentiation between BAT and WAT from the histology alone can be challenging. Further, due to small sample size limitation and inter individual variability of FAU in BAT, it is not possible to suggest a FAU value cutoff point for a subject to be likely possessing histologically evident BAT. The groups in this study were heterogenous and there was a difference in age, however not statistically significant difference, and a significant difference in M-value, which both correlated negatively with BAT FAU during cold exposure. However, in multiple linear regression analysis we did not find any confounding factors, besides age, which might contribute to the differences between lean and obese subjects. When comparing cold exposure and room temperature values we have performed paired t-tests, to reduce the effect of possible confounding factors. Additionally, BAT FAU measured with PET merely tells us the tissue intake of fatty acids, and this method cannot reveal subsequent post-uptake fate of fatty acids i.e. whether they are utilized for mitochondrial oxidative metabolism or intracellular lipid storage.

In conclusion, we show here that BAT FAU and perfusion increased after cold exposure in lean, but not in obese subjects. Lean subjects have twofold higher BAT FAU during acute cold exposure, and at basal state in room temperature, compared to obese subjects. Subjects with histological evidence of BAT manifest a threefold increase in BAT FAU in response to cold, and these subjects have fourfold higher FAU in BAT during cold stress compared to other lean subjects with no histologically evident BAT, and sevenfold greater FAU compared to obese subjects with no histological evidence of BAT. The subjects with greater FAU in BAT are leaner, younger and have better whole-body insulin sensitivity. This suggests that the potential role of BAT for thermoregulation and/or for excess energy disposal is impaired in obesity both at basal and cold-activated state.

## Methods

We recruited both lean and obese subjects of both genders. Obesity was defined as BMI ≥ 30 kg/m^2^. All subjects were metabolically healthy as judged by medical history (anamnesis), screening laboratory tests and oral glucose tolerance test (OGTT). OGTT results were additionally used to calculate Matsuda index, HOMA-IR and insulinogenic index^30,31^. After screening each study subject underwent a PET/CT imaging session during acute cold exposure. Another imaging session on a separate day was performed in ambient room temperature for 22 (16 lean and 6 obese) subjects (Fig. 2). The order in which cold and room temperature imaging was performed was random. All the imaging sessions were conducted at the same time of the day to avoid any influence of individual-circadian rhythm. Grouping of the study participants are given in shown in Fig. 2. All studies were carried out after overnight fasting of at least 12 h. Venous blood samples were drawn to measure tracer metabolites and several other variables. Hyperinsulinemic euglycemic clamp study was performed to measure whole-body insulin sensitivity (M-value)^32^.

The study protocol was reviewed and approved by the Ethics Committee of the Hospital District of Southwest Finland and the study was carried out according to the principles of the Declaration of Helsinki and GCP guidelines. Written informed consent was signed by all study subjects prior to any study procedures and inclusion in the study.

### PET scans

General study outline is shown in Fig. 1. All scans were performed with the same PET/CT scanner (Discovery 690 PET-CT scanner; General Electric Medical Systems, Milwaukee, WI, USA). Individualized cold exposure started 2 h before the scanning session and continued throughout the scanning session. Cooling blankets with flowing water inside were used to achieve the personal non-shivering state of the subject. The temperature of the cooling blankets was adjustable, and the temperature was adjusted to avoid shivering. The average temperature of the water inside the blanket at the start of the scan was 15.0 ± 2.0 °C for the lean group and 10.0 ± 4.8 °C for the obese group.

The room temperature scans were performed identically to the cold exposure scans, except with the absence of cooling blankets. Room temperature during these control scans was 22.3 ± 0.5 °C.

Tissue specific perfusion was quantified with an intravenous injection of ^15^O-H_2_O and a dynamic emission scan of the thoracic region was performed (frames: 6 × 5 s, 6 × 15 s, 8 × 30 s). Radiowater was produced using Hidex Radiowater Generator (Hidex Oy, Turku, Finland).

To quantify FAU we used 14(*R,S*)-^18^F-fluoro-6-thia-heptadecanoic acid, ^18^F-FTHA, a palmitate analogue. After ^15^O-H_2_O had sufficiently decayed (i.e. 10 min), an intravenous injection of ^18^F-FTHA was given and a dynamic emission scan was performed (frames: 1 × 60 s, 6 × 30 s, 1 × 60 s, 3 × 300 s, 2 × 600 s) on the same area as with radiowater. ^18^F-FTHA was produced as previously described^33,34^.

After acquisition of scanning data computerized reconstruction was carried out. Quantitative corrections were applied to PET image data, including detector normalization, dead-time, radioactive decay, randoms, attenuation and scatter. Images were reconstructed using iterative 3D-OSEM (GE Vue Point HD-S) reconstruction using 24 subsets and 2 iterations. All images were filtered using 6.4 mm Gaussian post-filter.

### PET data analysis

Volumes of interest (VOI) were drawn manually using fused dynamic PET-CT images. VOIs were drawn in the supraclavicular fat depots (typical BAT area), the lateral part of the deltoid muscles and subcutaneous white adipose tissue (WAT) in the posterior area of the neck. Input functions for modeling calculations were derived from the images by drawing a VOI into the arch of the aorta. Tissue specific FAU was calculated using multiple time graphical analysis^35–37^. Plasma input functions were corrected for metabolites which were measured from plasma samples acquired during the scanning. Net influx rate (K_i_) of ^18^F-FTHA from the graphical analysis was multiplied by plasma FFA concentrations measured during the scans to calculate tissue specific FAU.

Tissue specific perfusion was calculated using the 1-tissue compartment model, a method based on the principle of exchange of inert gas between blood and tissues^38^. Input functions were derived from the images, by drawing a VOI in the arch of the aorta. The same VOIs used to acquire TACs in FAU calculations were used in perfusion analysis.

All analysis of PET-CT images was done using Carimas software (Turku PET Centre, Turku, Finland)^39–41^.

### Statistical analysis

Power calculations and number of subjects was determined based on previous studies using ^18^F-FDG and previous unpublished ^18^F-FTHA data^3,6,7^. Statistical analyses were performed using IBM SPSS Statistics (version 22). Comparison of means was performed with two-way Student’s t-test (lean vs. obese; unpaired t-test; BAT^(+)^ vs. BAT^(−)^; unpaired t-test; room temperature vs. cold: paired t-test, or as otherwise stated). Associations between variables were calculated using Pearson’s, Spearman’s rank correlation and point-biserial correlation when correlating categorical and continuous variables. Multiple linear regression analysis was performed to further evaluate correlations between variables and to adjust for confounding factors. P-value of less than 0.05 was considered as statistically significant. Data is presented as mean ± SD, unless stated otherwise. N represents number of subjects; details of groups and statistical parameters can be found in the figure legends.

### Adipose tissue biopsies

Biopsies were obtained from the supraclavicular region of 31 subjects. Site of the biopsy was determined by imaging data of the area (^18^F-FTHA PET/CT or MRI). The procedure was carried out by a plastic surgeon at room temperature (~ 22 °C) under local anesthesia. Subcutaneous WAT sample was collected from the same incision. The samples were fixed in formalin after extraction^3^. BAT was defined based on morphology by an experienced pathologist, and samples designated as BAT positive showed cells with multilocular lipid droplets.

## Supplementary information

Supplementary Information
