# Supplementary material for: Basal and cold-induced fatty acid uptake of human brown adipose tissue is impaired in obesity
Source: Sci Rep. 2020 Sep 1;10:14373. doi: 10.1038/s41598-020-71197-2 (PMC7463032; doi:10.1038/s41598-020-71197-2)
Supplement: Supplementary file 1 — Supplementary Information [file 41598_2020_71197_MOESM1_ESM.pdf]

Basal and cold-induced fatty acid uptake of human brown adipose tissue is impaired in obesity

Saari TJ[1,2], Raiko J[2], U-Din M[1, 2], Niemi T[3], Taittonen M[4], Laine J[5], Savisto N[2], Haaparanta-Solin M[2,6], Nuutila P[1, 2], Virtanen KA[1, 2, 7,*]

[1] Turku PET Centre, Turku University Hospital, Kiinamyllynkatu 4-8, 20520, Turku, Finland

[2] Turku PET Centre, University of Turku, Kiinamyllynkatu 4-8, 20520, Turku, Finland

[3] Department of Surgery, Turku University Hospital, Kiinamyllynkatu 4-8, 20520, Turku, Finland

[4] Department of Anesthesiology, Turku University Hospital, Kiinamyllynkatu 4-8, 20520 Turku, Finland

[5] Department of Pathology, Turku University Hospital, Kiinamyllynkatu 4-8, 20520, Turku, Finland

[6] MediCity Research Laboratories, University of Turku, Tykistökatu 6A, 20520, Turku, Finland

[7] Institute of Public Health and Clinical Nutrition, University of Eastern Finland, PL 1627, 70211, Kuopio, Finland

* Corresponding author

Corresponding author contact information:
Kirsi A. Virtanen, MD, PhD
Adjunct professor/Docent
Turku PET Centre
Turku University Hospital and University of Turku
Kiinamyllynkatu 4-8
20520 Turku
mobile +358 40 7626564
email: kirsi.virtanen@utu.fi or kirsi.virtanen@tyks.fi

Supplementary Table 1. General characteristics of histologically proven BAT positive (BAT$^{(+)}$) and BAT negative subjects (BAT$^{(-)}$) and measurements taken at baseline, and during cold exposure or room temperature scans. Values are presented as mean ± SD.

		**Histologically evident BAT-positive n = 7**	**Histologically evident BAT-negative n = 24**	**p-value**
Age (years)		27.57 ± 7.7	40.52 ± 10.68	0.004
Gender (M/F)		4F / 3M	10F / 11M	
BMI (kg/m^2)		24.8 ± 3.0	28.11 ± 5.05	0.050
Waist circumference (cm)		85.71 ± 10.87	94.98 ± 18.86	0.13
M-value (mg/kg/min)		7.35 ± 2.14	6.34 ± 3.92	0.40
Fat percentage of weight (%)		30.26 ± 9.37	30.55 ± 8.60	0.5
HDL cholesterol (mmol/L)		1.74 ± 0.42	1.49 ± 0.39	0.207
LDL cholesterol (mmol/L)		2.71 ± 0.95	2.84 ± 0.67	0.76
Total cholesterol (mmol/L)		4.57 ± 0.88	4.82 ± 0.72	0.526
Triglycerides (mmol/L)		0.81 ± 0.21	1.05 ± 0.71	0.205
HbA_{1c} (mmol/mol)		33.19 ± 4.21	35.47 ± 4.50	0.248
Alanine transaminase (U/L)		19.57 ± 10.39	29.15 ± 15.50	0.086
Gamma-glutamyltransferase (U/L)		15.86 ± 7.82	27.45 ± 19.22	0.036
Alkaline phosphatase (U/L)		49.0 ± 17.04	64.10 ± 17.73	0.065
**COLD EXPOSURE**	BAT Fatty Acid Uptake (µmol/100g/min)	2.64 ± 1.01	0.54 ± 0.24	0.001
	BAT blood flow (mL/100g/min)	16.56 ± 5.80	11.06 ± 4.96	0.051
	BAT radiodensity (HU)	-82.85 ± 5.64	-88.25 ± 9.72	0.089
	WAT Fatty Acid Uptake (µmol/100g/min)	0.31 ± 0.12	0.22 ± 0.067	0.079
	WAT blood flow (mL/100g/min)	2.44 ± 1.22	2.84 ± 0.96	0.45
	WAT radiodensity (HU)	-83.99 ± 2.88	-83.12 ± 6.88	0.64
	Deltoid Fatty Acid Uptake (µmol/100g/min)	0.76 ± 0.22	0.44 ± 0.13	0.007
	Deltoid blood flow (mL/100g/min)	4.10 ± 2.84	3.04 ± 2.97	0.416
	Deltoid radiodensity (HU)	64.44 ± 14.51	37.49 ± 34.03	0.008
**ROOM TEMPERATURE**	BAT Fatty Acid Uptake (µmol/100g/min)	1.19 ± 0.43	0.36 ± 0.22	0.025
	BAT blood flow (mL/100g/min)	6.38 ± 4.25	7.02 ± 4.74	0.83
	BAT radiodensity (HU)	-86.46 ± 5.97	-89.89 ± 9.38	0.28
	WAT Fatty Acid Uptake (µmol/100g/min)	0.32 ± 0.13	0.22 ± 0.10	0.22
	WAT blood flow (mL/100g/min)	1.95 ± 0.44	3.52 ± 3.80	0.19
	WAT radiodensity (HU)	-84.41 ± 0.91	-83.80 ± 5.45	0.70
	Deltoid Fatty Acid Uptake (µmol/100g/min)	0.53 ± 0.034	0.40 ± 0.11	0.003
	Deltoid blood flow (mL/100g/min)	2.22 ± 1.85	2.10 ± 0.89	0.9
	Deltoid radiodensity (HU)	52.44 ± 17.20	38.02 ± 29.31	0.13
**All data presented as mean ± SD**<br>Unpaired student t-test has been used to compare data between lean and obese study subjects				

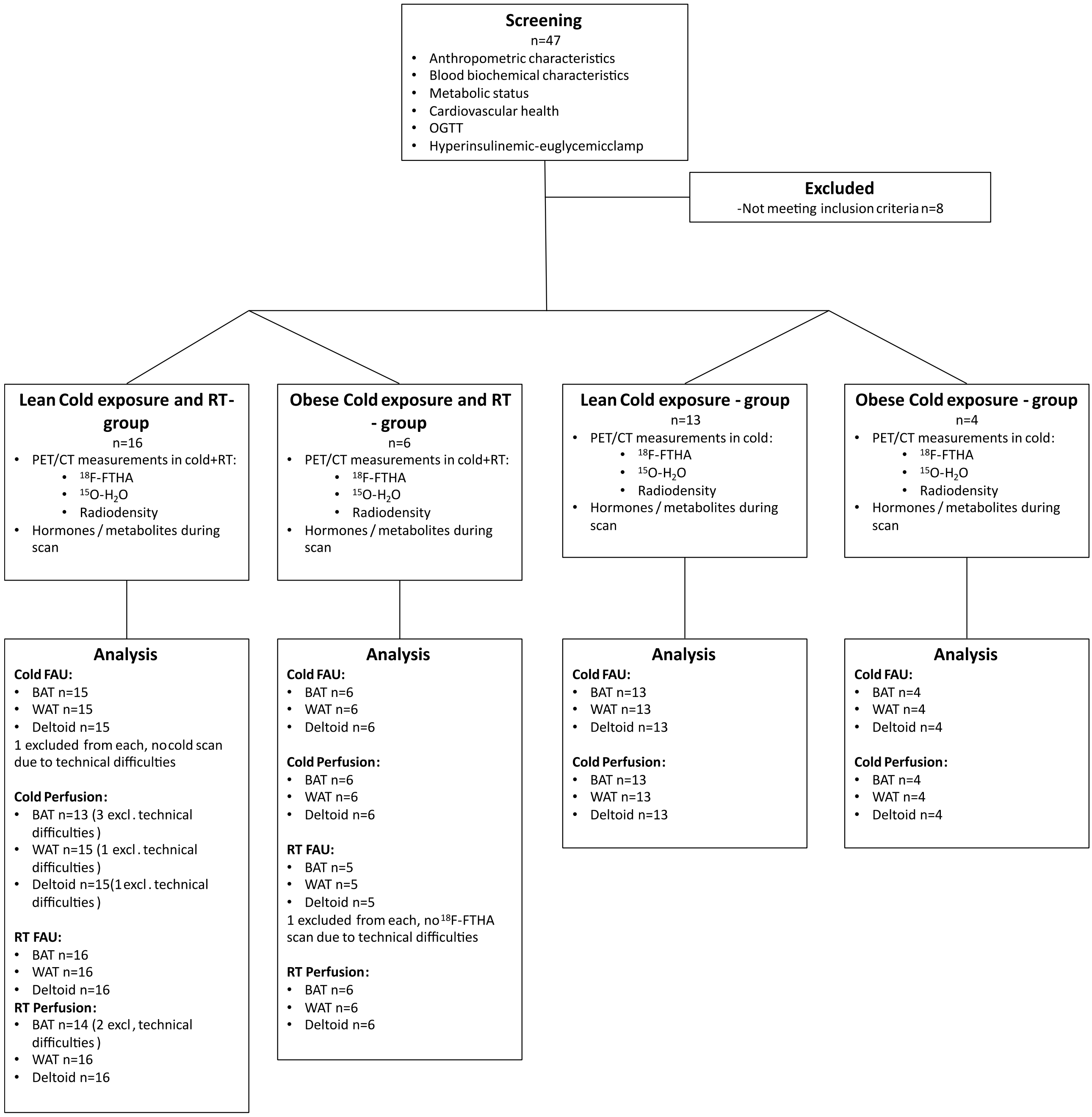

Supplementary Figure 1. More detailed version of Figure 2, with all excluded PET measurement details.
